# Prediction of Membrane Failure in a Water Purification Plant Using Nonhomogeneous Poisson Process Models

**DOI:** 10.3390/membranes11110800

**Published:** 2021-10-20

**Authors:** Takashi Hashimoto, Satoshi Takizawa

**Affiliations:** 1 Research Center for Advanced Science and Technology, the University of Tokyo, 4-6-1 Komaba, Meguro-ku, Tokyo 153-8904, Japan; 2 Department of Urban Engineering, the University of Tokyo, 7-3-1 Hongo, Bunkyo-Ku, Tokyo 113-8656, Japan; takizawa@env.t.u-tokyo.ac.jp

**Keywords:** membrane filtration, membrane failure, nonhomogeneous Poisson process, bootstrap, module replacement

## Abstract

The prediction of membrane failure in full-scale water purification plants is an important but difficult task. Although previous studies employed accelerated laboratory-scale tests of membrane failure, it is not possible to reproduce the complex operational conditions of full-scale plants. Therefore, we aimed to develop prediction models of membrane failure using actual membrane failure data. Because membrane filtration systems are repairable systems, nonhomogeneous Poisson process (NHPP) models, i.e., power law and log-linear models, were employed; the model parameters were estimated using the membrane failure data from a full-scale plant operated for 13 years. Both models were able to predict cumulative failures for forthcoming years; nonetheless, the power law model showed higher stability and narrower confidence intervals than the log-linear model. By integrating two membrane replacement criteria, namely deterioration of filtrate water quality and reduction of membrane permeability, it was possible to predict the time to replace all the membranes on a water purification plant. Finally, the NHPP models coupled with a nonparametric bootstrap method provided a method to select membrane modules for earlier replacement than others. Although the criteria for membrane replacement may vary among membrane filtration plants, the NHPP models presented in this study could be applied to any other plant with membrane failure data.

## 1. Introduction

Membrane filtration systems have been widely applied to water purification, including household-level systems and wastewater reuse [1,2]. Among the various types of membranes, hollow fiber membranes are widely used for water purification because of larger surface areas and high filtration performances. However, the integrity loss associated with membrane failure is of considerable concern [3,4] because it compromises the safety of the filtrate due to contamination by pathogenic microorganisms in unfiltered bypass-flow water [5,6]. 

To ensure treated water safety in membrane filtration plants, integrity testing of membrane modules is conducted on- or offline [5,7]. There are two types of integrity testing: direct integrity testing based on detecting the fiber failure by offline pressure-based tests [8,9], and indirect integrity testing based on monitoring the change in filtrate quality during operation [10,11]. Although direct integrity testing has higher sensitivity in detecting membrane integrity loss than indirect integrity testing [5], filtration operation must be suspended to perform direct integrity testing. Thus, there is a delay in detecting membrane failure from the time when it actually happened, which results in the leakage of raw water into the filtrate [12].

Once the integrity loss is detected in a membrane module, a failed hollow fiber is repaired by plugging with a stainless-steel pin or epoxy adhesive [5,13]. To avoid the risks of filtrate water quality deterioration, a membrane module is replaced with a new one when the frequency of membrane failure increases [13]. The cost of membrane replacement is reported to be several to twenty percent of the total production costs [14,15]. Thus, it is necessary for waterworks to predict membrane failures to ensure water safety and to formulate their management plans [13,16], including operation and maintenance costs [17]. 

To estimate the time and number of membrane failures, lifetime prediction methods based on accelerated laboratory tests are commonly applied [18,19,20]. Generally, accelerated tests of membranes are performed by soaking membranes in high chemical concentrations and/or for a longer chemical contact time than actual conditions to simulate membrane degradation [21,22,23]. However, factors other than contact with cleaning chemicals also influence the membrane lifetime, such as fouling conditions, the number of backwashings applied, and their combination [21,24]. Thus, accelerated tests cannot predict membrane failure with high accuracy. Another limitation of accelerated tests is that the service time of the membrane module is not determined by the average lifetime of hollow fiber membranes; it is commonly determined by the filtration performance examined by integrity testing [5,13], which is influenced by a small number of membranes prone to failure. Thus, the service time of membrane modules could be more accurately predicted by actual data obtained from membrane filtration plants rather than by accelerated testing in a laboratory. 

The failure data of actual systems are used to predict the lifetimes of systems in other engineering fields, such as the occurrence rate of failures or the interval time between failures [25,26]. As a system is subject to complex deterioration mechanisms, the lifetime of a system using the inspection data is usually predicted with a statistical model that reflects the stochastic nature of deterioration and various uncertainties [26]. Several classes of statistical lifetime models exist, such as Weibull distribution models [27], Poisson process models [28], log-normal distribution [29], gamma distribution [30], or combinations of these models [31,32]. Among these models, homogeneous or nonhomogeneous Poisson processes (HPP or NHPP, respectively) are robust and have the advantage of being able to deal with discrete data, such as the number of membrane failures or the rate of occurrence of membrane failures; thus, they are most frequently applied to the failure or lifetime analysis of systems [28,33]. 

In membrane filtration systems, once a membrane failure is found, the failed membrane fiber is plugged to reinstate the membrane module to the operational state similar to one without membrane failure [5]. Thus, although a failed membrane fiber is not repairable, a membrane module and a membrane filtration system are repairable, which are commonly modeled by HPP or NHPP [34]. The degradation process of a membrane fiber is a time-dependent phenomenon, impacted by the environmental conditions and the system state, which usually change over time [26,35]. Therefore, NHPP is suitable for lifetime prediction of membrane filtration systems, as the intensity of NHPP is described as a function of time. NHPP is a model of HPP generalized by incorporating the change in the intensity as a function of time [34], and commonly applied to describe the lifetime modeling of engineering systems [26]. 

The rate of membrane degradation may also be influenced by the variation in membrane diameter and strength caused in manufacturing processes [36]. This indicates that the failure rates of hollow fiber membranes in membrane modules that comprise a membrane filtration plant vary due to two reasons: manufacturing variation and statistical deviation. Several NHPP models incorporate the heterogeneity of multiple systems. In the study of pipeline failure modeled using power law NHPP [37], the pipeline length factor was added to the intensity function of the model as a known scaling factor. As another example of failure studies on wind turbines or electrical equipment in a manufacturing plant, a trend function with additional covariates was introduced into the intensity function of NHPP models to identify the cause of heterogeneity in the failure trend [38,39]. However, these extensions of NHPP models cannot be applied to membrane failure processes, as the individual variation among membrane modules is unknown and there may be no proper covariate for membrane systems due to the identical environmental factors and operational and maintenance conditions.

Therefore, the application of these models to the prediction of membrane failure in a membrane filtration plant is limited, although there are a couple of studies on the lifetime modeling of the membrane by combining the accelerated test and a Weibull distribution model [18] or combining the bootstrapping method and the experimental polymer ageing model [19]. One of the reasons for this lack of studies on the prediction of membrane failure is that most of the membrane-based water purification systems have been installed relatively recently; thus, membrane failure data in actual plants have not yet been systematically collected and analyzed. Thus, it would provide useful information to verify the applicability of statistical models to the lifetime prediction of membrane modules and membrane filtration systems.

In this study, we aimed to construct a novel method to predict membrane failures using NHPP models. In this study, two NHPP models with different intensity functions, namely a power law model and a log-linear model, were employed to predict membrane failures in a water purification plant and in each membrane module. To reach a decision on membrane module replacement, a novel strategy based on two criteria, namely the membrane failure rate and the performance reduction due to both membrane fouling and fiber failure, was developed. The individual property variation of modules was incorporated into the performance reduction criterion by combining NHPP models and a bootstrapping method. 

## 2. Materials and Methods

### 2.1. Membrane Filtration Plant and Membrane Failure Detection

The membrane failure data were obtained from a small-scale membrane filtration plant in Japan (Table 1). The plant uses a polyacrylonitrile (PAN) ultrafiltration hollow fiber membrane with the molecular weight cut-off (MWCO) of 1,500,000 (Table 2). The membrane facility comprised a total of fifteen membrane modules (five modules/train × three trains). A train refers to a group of membrane modules that are operated in a unit; thus, they experience the same conditions over time. The raw water was taken from a river and fed to the system without coagulation. Physical cleaning such as air scrubbing and backwashing was conducted every forty-five minutes. Chemical cleaning with acid and hypochlorite was conducted every six to nine months.

The operational age of the membranes was 12.7 years at the time the operational data were obtained. Pressure-based integrity testing was implemented once or twice a year when the offline chemical cleaning was conducted. Once a membrane failure was detected, the damaged hollow fiber membranes were repaired by plugging with a stainless-steel pin. During the course of membrane filtration operation, no membrane module was replaced.

### 2.2. Statistical Inference of Membrane Failure Process

#### 2.2.1. Membrane Failure Data

The number of failed hollow fiber membranes in each module was obtained when direct integrity testing was conducted in association with the offline chemical cleaning. Thus, the exact date of membrane failure of each module could not be identified, but recorded as failure events between two dates of integrity tests. Then, the yearly membrane failure was calculated by dividing the number of failed membranes by the duration of the two integrity tests in terms of years.

#### 2.2.2. Statistical Models for the Membrane Failure Process

Hollow fiber membranes are subjected to physical and chemical stress (e.g., chemical substances in raw water and repeated chemical cleaning) throughout their operation, and the rate of membrane failure may increase due to the progress of membrane degradation. Therefore, membrane failure models need to assume the instantaneous failure rate as a function of time, ν(t) [40]. In NHPP, the expected number of failures in the time interval (t,t+∆t] is denoted by $\int_{t}^{t+∆t}\nu \left(x\right)dx$, which is the probability that a failure will occur in the interval following a Poisson distribution with an intensity of $\int_{t}^{t+∆t}\nu \left(x\right)dx$.

The expected number of failures by the time t for NHPP is described as:

(1) $$E\left(N\left(t\right)\right)=\int_{0}^{t}\nu \left(x\right)dx$$

If the exact failure times are unknown and only the number of failures within a time interval are recorded, e.g., the number of membrane failures $n_{i}$ within a time interval $(t_{i-1},\;t_{i}]$ between the successive integrity tests, the grouped data approach is used for estimation of parameters [39]. The number of failures in a unit interval $(t_{i-1},\;t_{i}],\;i\in \left\{1,\cdots ,\;k\right\}$ follows a Poisson distribution with an intensity of $\int_{t_{i-1}}^{t_{i}}\nu \left(s\right)ds$. The joint probability of *n_i_* failures in interval $(t_{i-1},\;t_{i}],\;i\in \left\{1,\cdots ,\;k\right\}$ is equal to the product of the probabilities in each interval:

(2) $$\begin{array}{l} P\left(N\left(t_{1}\right)-N\left(t_{0}\right)=I,\;\ldots ,\;N\left(t_{k}\right)-N\left(t_{k-1}\right)=n_{k}\right)\\ =\overset{k}{\underset{i=1}{\prod }}P\left(N\left(t_{i}\right)-N\left(t_{i-1}\right)=n_{i}\right)\\ =\overset{k}{\underset{i=1}{\prod }}\frac{{\left(\int_{t_{i-1}}^{t_{i}}\nu \left(s\right)ds\right)}^{n_{i}}\cdot exp\left(-\int_{t_{i-1}}^{t_{i}}\nu \left(s\right)ds\right)}{n_{i}!} \end{array}$$

The last equation in Equation (2) is the likelihood function.

In this study, two NHPP models, i.e., the power law model and the log-linear model, were investigated.

- Power law model

In the power law model, the expected number of cumulative failures is expressed as:

(3) $$\begin{array}{r} E\left(N\left(t\right)\right)=\lambda t^{\beta }\\ (0<\;\lambda ,\;\beta ,\;\mathrm{and}\;0<t) \end{array}$$

where λ is the scale parameter, β is the growth parameter determining improvement or deterioration over time, and t is the system operation time. 

The cumulative failure rate $v_{c}$ and the cumulative mean time between failure ($MTBF_{c}$) are respectively described by:

(4) $$v_{c}=\frac{E\left(N\left(t\right)\right)}{t}=\lambda t^{\beta -1}$$

(5) $$MTBF_{c}=\frac{1}{\lambda }t^{1-\beta }$$

The instantaneous failure rate at time t, or intensity function $\nu_{pp}\left(t\right)$, is described by:

(6) $$\nu_{pp}\left(t\right)=\lambda \beta t^{\beta -1}$$

For 1<β, the failure rate increases. For β<1, the failure rate decreases. For β=1, the failure rate is constant, which reverts NHPP to HPP. 

For estimation of parameters λ and β, the maximum likelihood estimation method was applied with the likelihood function:

(7) $$L=\overset{k}{\underset{i=1}{\prod }}\frac{{\left(\lambda t_{i}{}^{\beta }-\lambda t_{i-1}{}^{\beta }\right)}^{n_{i}}\cdot \exp\left(-\left(\lambda t_{i}{}^{\beta }-\lambda t_{i-1}{}^{\beta }\right)\right)}{n_{i}!}$$

(8) $$\log L=-\lambda T_{k}{}^{\beta }+\overset{k}{\underset{i=1}{\sum }}\left[n_{i}\log\left(\lambda t_{i}{}^{\beta }-\lambda t_{i-1}{}^{\beta }\right)-\log\left(n_{i}!\right)\right]$$

where $T_{k}$ is the failure time for the *k*-h failure event.

Maximum likelihood estimators (MLEs) of λ and β were computed from Equation (8) via a quasi-Newton method algorithm with the R package ‘bbmle’ [41]. The Duane model (Equation (9) [42]) was used to obtain the starting values for the quasi-Newton method:

(9) $$\log\left(MTBF_{c}\right)=-\log\lambda +\left(1-\beta \right)\log t$$

- Log-linear model

In the log-linear model, the intensity function $\nu_{ll}\left(t\right)$ is described as:

(10) $$E\left(N\left(t\right)\right)=\frac{e^{\left(\gamma_{0}+\gamma_{1}t\right)}}{\gamma_{1}}$$

(11) $$\nu_{ll}\left(t\right)=\exp\left(\gamma_{0}+\gamma_{1}t\right)$$

where $\nu_{ll}\left(t\right)$ is the instantaneous failure rate, $\gamma_{0}$ is the scale parameter, $\gamma_{1}$ is the growth parameter determining improvement or deterioration over time, and t is the system operation time. 

The likelihood function for the log-linear model is described as:

(12) $$L=\overset{k}{\underset{i=1}{\prod }}\left\{\left(\frac{1}{n_{i}!}\right){\left(\frac{e^{\gamma_{0}+\gamma_{1}t_{i}}-e^{\gamma_{0}+\gamma_{1}t_{i-1}}}{\gamma_{1}}\right)}^{n_{i}}\cdot \exp\left(-\left(\frac{e^{\gamma_{0}+\gamma_{1}t_{i}}-e^{\gamma_{0}+\gamma_{1}t_{i-1}}}{\gamma_{1}}\right)\right)\right\}$$

(13) $$\log L=\overset{k}{\underset{i=1}{\sum }}\left[-\log\left(n_{i}!\right)+n_{i}\left\{\log\left(\frac{e^{\gamma_{0}+\gamma_{1}t_{i}}-e^{\gamma_{0}+\gamma_{1}t_{i-1}}}{\gamma_{1}}\right)\right\}-\left(\frac{e^{\gamma_{0}+\gamma_{1}t_{i}}-e^{\gamma_{0}+\gamma_{1}t_{i-1}}}{\gamma_{1}}\right)\right]$$

Parameters $\gamma_{0}$ and $\gamma_{1}$ were estimated by the maximum likelihood estimation method via a quasi-Newton method algorithm with the R function ‘optim’ for general-purpose optimization.

The confidence interval ((1−α)×100%) for cumulative number of failures N(t) for power law model or log-linear model is expressed as:

(14) $$N\left(t\right)=\hat{N}\left(t\right)exp\left(\pm z_{\alpha }\sqrt{\frac{Var\left(\hat{N}\left(t\right)\right)}{\hat{N}\left(t\right)}}\right)$$

where $\hat{N},\;\hat{\lambda },\;\hat{\;\beta },\;{\hat{\;\gamma }}_{0}$, and ${\hat{\gamma }}_{1}$ represent the estimated values of N, λ, β, $\gamma_{0}$, and $\gamma_{1}$, respectively.

$$\begin{array}{l} \left(\mathrm{for}\;\mathrm{the}\;\mathrm{power}\;\mathrm{law}\;\mathrm{model}\right)\\ \hat{N}\left(t\right)=\hat{\lambda }t^{\hat{\beta }}\\ Var\left(\hat{N}\left(t\right)\right)={\left(\frac{\partial \hat{N}\left(t\right)}{\partial \beta }\right)}^{2}Var\left(\hat{\beta }\right)+{\left(\frac{\partial \hat{N}\left(t\right)}{\partial \lambda }\right)}^{2}Var\left(\hat{\lambda }\right)+2\left(\frac{\partial \hat{N}\left(t\right)}{\partial \beta }\right)\left(\frac{\partial \hat{N}\left(t\right)}{\partial \lambda }\right)Cov\left(\hat{\beta },\;\hat{\lambda }\right) \end{array}$$

$$\begin{array}{l} \left(\mathrm{for}\;\mathrm{the}\;\log\text{-}\mathrm{linear}\;\mathrm{model}\right)\\ \hat{N}\left(t\right)=\frac{e^{\left({\hat{\gamma }}_{0}+{\hat{\gamma }}_{1}t\right)}}{{\hat{\gamma }}_{1}}\\ Var\left(\hat{N}\left(t\right)\right)={\left(\frac{\partial \hat{N}\left(t\right)}{\partial \gamma_{0}}\right)}^{2}Var\left({\hat{\gamma }}_{0}\right)+{\left(\frac{\partial \hat{N}\left(t\right)}{\partial \gamma_{1}}\right)}^{2}Var\left({\hat{\gamma }}_{1}\right)+2\left(\frac{\partial \hat{N}\left(t\right)}{\partial \gamma_{0}}\right)\left(\frac{\partial \hat{N}\left(t\right)}{\partial \gamma_{1}}\right)Cov\left({\hat{\gamma }}_{0},\;{\hat{\gamma }}_{1}\right) \end{array}$$

#### 2.2.3. Estimation of Model Parameter Distribution by the Bootstrap Method

A nonparametric bootstrap method was applied for the statistical inference of the parameters of each NHPP model per module. From the original data set of model parameter $X=\left\{x_{i},\;i=1,\;2,\;\ldots ,\;N\right\}$, a bootstrap sample set $B_{j}=\left\{B_{1,j},\;B_{2,j},\;\ldots B_{N,j}\right\}$ was generated by random sampling with replacement from X. Here, j is the total number of iterations of sampling. With the generated bootstrap sample set $B_{j}$, the set of sample median $B_{j}^{md}$ was obtained. The mean and the variance of $B_{j}^{md}$ are given by:

(15) $${\bar{B}}_{N,j}^{md}=\frac{1}{N}\overset{N}{\underset{i=1}{\sum }}B_{i,j}^{md}$$

(16) $$Var\left(B_{N,j}^{md}\right)=\frac{1}{N-1}\overset{N}{\underset{i=1}{\sum }}{\left(B_{i,j}^{md}-{\bar{B}}_{N,j}^{md}\right)}^{2}$$

The bootstrap method was applied to the estimated model parameters per module, $\lambda_{i}$, $\beta_{i}$, $\gamma_{0,i}$, and $\gamma_{1,i}$ (where i is the module number). The mean and the variance of bootstrapped samples for each parameter were obtained by Equations (15) and (16), respectively. The covariance of $\lambda_{i}$ and $\beta_{i}$, or $\gamma_{0,i}$, and $\gamma_{1,i}$ is given by:

(17) $$cov\left(B_{N,j}^{md}\right)=\frac{1}{N-1}\overset{N}{\underset{i=1}{\sum }}{\left(B_{i,j}^{md}-{\bar{B}}_{N,j}^{md}\right)}^{2}$$

The confidence interval ((1−α)×100%) for each NHPP model with bootstrapped parameters was obtained from Equation (14).

### 2.3. Requirement for Membrane Filtration Performance

It is expected that, in membrane filtration processes, the removal rates of suspended solids, bacteria, and protozoa meet certain criteria [13]. In this study, the required removal rate for microfiltration membranes was set by the logarithmic reduction value (LRV) based on the USEPA’s LT2ESWTR [43]. The LT2ESWTR requires a *Cryptosporidium* removal of at least 4-log for the entire water purification process, and a minimum removal of 2-log for the filtration process [43]. Thus, we adapted a 2-log removal as a minimum requirement for the membrane filtration process even if some of the hollow fiber membranes failed.

The LRV of a failed membrane module was calculated from Equation (18) based on Liu [5]:

(18) $$LRV=\log_{10}\frac{q_{f}\left(n-n_{f}\right)+q_{b}n_{f}}{q_{b}n_{f}}$$

where $q_{f}$ is the flow rate through an intact hollow fiber membrane $\left(m^{3}\;s^{-1}\right)$, $q_{b}$ is the bypass flow rate through a failed hollow fiber membrane $\left[m^{3}\;s^{-1}\right]$, n is the total number of hollow fiber membranes in a module, and $n_{f}$ is the number of failed hollow fibers.

The flow rate through an intact hollow fiber membrane $q_{f}$ was calculated from Equation (19):

(19) $$q_{f}=\frac{J\times A}{86400}=\frac{J\times d_{out}\times \pi \times L}{86400}$$

where J is the average filtration flux of the system $\;\left(0.85\;m^{3}\;m^{-2}\;d^{-1}\right)$, A is the surface area of an intact hollow fiber membrane ($m^{3}$), $d_{out}$ is the outer diameter of hollow fiber $\left(1.4\times 10^{-3}\;m\right)$, and L is the effective length of the intact fiber (1.9 m).

A bypass flow rate was calculated as follows: the equation for a bypass flow rate was determined by the flow regime in a hollow fiber, which can be identified by Reynold’s number Re:

(20) $$Re=\frac{\rho d_{in}v}{\mu }=\frac{\rho d_{in}{}^{3}\Delta P}{32\mu^{2}l}$$

where ρ is the density of water (kg m^−3^), $d_{in}$ is the inner diameter of a hollow fiber $\;(0.8\times 10^{-3}\;m$, v is the average flow rate in a hollow fiber lumen ($m\;s^{-1}$), μ is the viscosity of water (0.00101 Pa s at 20 °C), ΔP is the transmembrane pressure ($10^{3}\;\mathrm{Pa}$), and l is the effective length of the broken fiber (m).

When Re<2000, the flow regime is laminar flow, which can be assumed to be the case that the fiber breakage occurred on the opposite side of the filtrate outlet of the membrane module being operated by the outside-to-inside operation mode of the hollow fiber membrane. Then, the bypass flow rate $q_{b}$ follows the Hagen–Poiseuille flow:

(21) $$q_{b}=\frac{\pi d_{in}{}^{4}\Delta P}{128\mu l}$$

When Re>3000, the flow regime is turbulent flow, which occurs when the fiber breakage occurs near the filtrate outlet of the membrane module. Then, $q_{b}$ is described by the following equation [5]:

(22) $$q_{b}=0.718\pi {\left(\frac{\Delta P}{l}\right)}^{0.571}\frac{D^{2.714}}{\rho^{0.429}\mu^{0.143}}$$

The maximum number of failed fibers per year to meet LRV 2 is calculated to be 22 fibers per module or 330 fibers per plant from Equation (18). 

When a membrane failure is found by the integrity test, the failed fiber is plugged and not used for the rest of operation; thus, the number of intact hollow fiber membranes and membrane surface area for filtration decrease. Furthermore, the permeability of the membrane reduces due to the chemically irreversible membrane fouling as the filtration operation proceeds. Reductions in the effective surface area and the permeability require a higher transmembrane pressure, which may necessitate replacement of the membrane module.

The reduced water production by module at the operation time t (years), W(t), is expressed as the ratio against the initial filtration performance W(0):

(23) $$\frac{W\left(t\right)}{W\left(0\right)}={\left(\frac{J_{t}}{J_{t-1}}\right)}^{t}\times \frac{n-n_{f}}{n}\;∴\;n_{f}=n\left\{1-{\left(\frac{1}{0.965}\right)}^{t}\times \frac{W\left(t\right)}{W\left(0\right)}\right\}$$

where $J_{t}$ is the average flux of the membrane module at a designated pressure measured after $\left(t\;\left(year\right)/f_{c}\left(year/time\right)\right)$ th times of chemical cleaning, and $f_{c}$ is the chemical cleaning interval. In this study, $J_{t}/J_{t-1}$ was calculated to be 0.965 from the reported permeability data, indicating about a 30% reduction after ten years. The numbers of failed fibers, $n_{f}$, for W(t)/W(0) of 0.5, 0.6, and 0.7 were calculated from Equation (23).

## 3. Results

### 3.1. Membrane Fiber Failure in the Water Purification Plant

**Figure 1:**
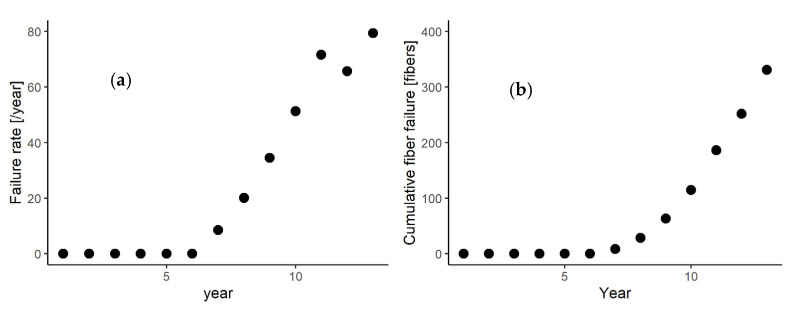
(**a**) Failure rate per year and (**b**) cumulative fiber failure of the plant.

The failure rate, i.e., the number of membrane failure per year, is shown in Figure 1a. The first membrane failure was observed in the seventh year of the operation, and then the failure rate increased thereafter. The increasing trend in the membrane failure rate is reflected by the increasing curve of cumulative membrane failure shown in Figure 1b. These results indicate that membrane failure is a nonhomogeneous process, which requires nonhomogeneous models such as NHPP. 

**Figure A1:**
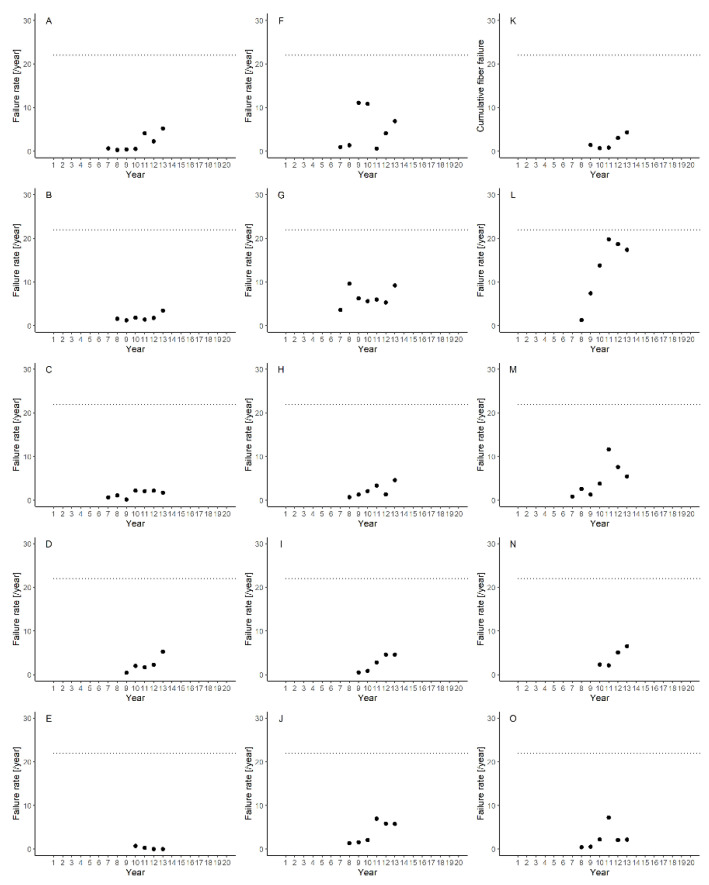
Failure rate (failure per year) for each membrane module. Train 1: (**A**–**E**); Train 2: (**F**–**J**); and Train 3: (**K**–**O**). Dotted lines indicate the permissible failure rate for LRV 2 (black).

Although there was a decrease in the failure rate in the 11th year from the 10th year, it increased again in the 12th year (Figure 1a). This kind of failure rate variation could be due to the variability in the failure rate for each membrane module, as observed in Figure A1. Although most of the membrane failure was detected in the eighth year, the increasing trend in the failure rate for each module significantly differed. For instance, a low failure rate was observed in Module E, but sharp increases in the failure rate were observed for Modules L and M. However, even with the large variability in the increasing trend, the failure rate of each module increased yearly due to membrane ageing [18]. These data indicate that, for predicting membrane failure, it is important to consider the variability in failure rate for each module.

### 3.2. Application of Nonhomogeneous Poisson Process Models to Membrane Failure

The power law model and the log-linear model of NHPP were applied to fit the membrane failure data.

#### 3.2.1. Membrane Failure Rates in the Water Purification Plant

- Power law model

**Figure 2:**
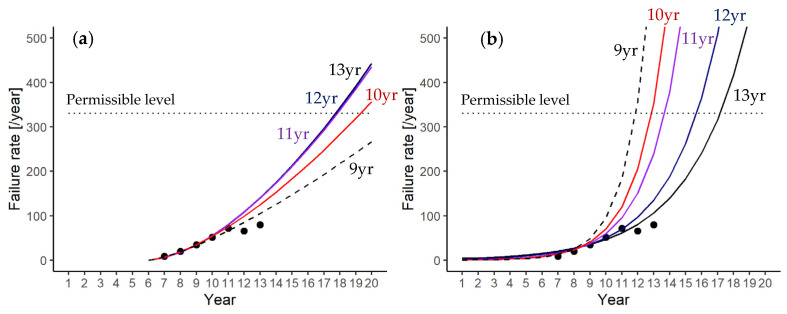
Membrane failure rates in the plant estimated by the power law model (**a**) and by the log-linear model (**b**). Dotted horizontal lines show the permissible failure rates: 330 (black) fibers per year.

The membrane failure rate curves for the whole plant estimated by the power law model using the failure data are shown in Figure 2a. Each line shows the failure rate estimated from the failure rate data until the operation year denoted in the figure. For example, the failure rate curve of the ninth year (9yr) was estimated from the failure data up to the ninth year of operation. The trends of these curves are influenced by the number of failure data obtained for each operation year. The rate curve drawn for the nine-year operation data in Figure 2a is the lowest, whereas the failure rate curves estimated from 10, 11, 12, and 13 years of operation gradually shift upward and then converge after the 11th year. The variation among them is less than 100 failures per year (about 30%) in the 16th year of operation. 

The predicted failure rates using the data up to the 10th year (10 yr) of operation exceeded the permissible failure rate (330 failures per year) after the 20th year of operation. The failure rates predicted from the data up to the 11th (11 yr), 12th (12 yr), and 13th (13 yr) years exceeded 330 after the 18th year of operation. Thus, the power law model predicts that the plant could be operated up to the 17th year without violating the criterion (LRV ≥ 2). As shown in this case, the power law model can predict the years of operation without violating the criterion with a small margin of error when it is applied for the membrane failure rate analysis of the whole plant.

- Log-linear model

The membrane failure rate curves for the whole plant estimated by the log-linear model are plotted in Figure 2b, which show abrupt increases compared to those by the power law model (Figure 2a). The failure rate curves by the log-linear model overestimated the permissible failure rate of 330 fibers per year at least up to the 11th year of operation, as shown by the wide gaps between the predicted and actual numbers of failure. This tendency of overestimation by the log-liner model may be due to the intensity function described by an exponential function.

#### 3.2.2. Cumulative Membrane Failure in the Water Purification Plant

- Power law model

**Figure 3:**
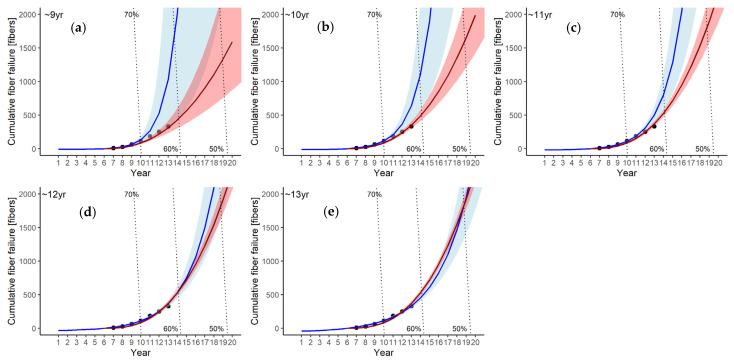
Predicted cumulative fiber failure by power law model (red) and log-linear model (blue). (**a**) predicted using data up to the 9th year; (**b**) 10th year; (**c**) 11th year; (**d**) 12th year; and (**e**) 13th year of operation. The shaded area shows the 95% confidence interval for each model. Dotted lines show cumulative failures for the reduced filtration performance level of 70%, 60%, and 50%.

The predicted cumulative failure curves using data up to the 9th to 13th years of operation are shown in Figure 3a–e. These predicted curves are similar within a small range except for the curve estimated using data only up to the ninth year (Figure 3, red lines). The 95% confidence intervals of the cumulative fiber failure predicted by the power law model are also shown in Figure 3 (red shaded area). All predicted curves fit the actual failures well (Figure 3a–d), and their root mean square errors (RMSEs) are within a small range (22.0–26.9, Table 3). As the number of failure data used for the prediction increases, the confidence intervals narrow. This suggests that the failure prediction performance is improved by adding more membrane failure data up to the 13th year of operation, which is indicative of the NHPP. This result is also in agreement with the Akaike’s information criterion (AIC) values, which decrease as the years of the failure data acquisition increase (Table 3).

The dotted lines in Figure 3 show the percentage reduction in the filtration performance associated with both membrane fouling and reduction in the membrane surface areas. From the crossing point between the reduction lines of the filtration performance and the predicted cumulative failure curve using 13 years of failure data, the membrane modules could be used until the 14th year if a 40% reduction in the filtration performance is assumed to be permissible. Moreover, if a 50% performance reduction is permissible, the membrane modules could be used until the 18th year.

- Log-linear model

The cumulative failure curves predicted by the log-linear model are shown by the blue lines in Figure 3a–e. The cumulative fiber failure curves predicted using the data up to the 9th, 10th, or 11th years (Figure 3a–c, respectively) show significant discrepancies from the actual numbers of failures. However, the predicted curves using the data up to the 12th or 13th years are closer to those predicted by the power law model. The 95% confidence intervals became narrower as the years of failure data used for the prediction increased. The AIC values for log-linear model decrease with the increase in the data acquisition period for prediction (Table 3). However, they are larger than those for power law model for all cases, which indicates the better prediction performance of the power law model than the log-linear model. The RMSE values of the log-linear models predicted from the data up to 12th and 13th year are smaller than those of the power law models (Table 3), which indicates that log-linear model better fits the observed data than the power law model when the number of data increases. These findings suggest that, for the prediction by the log-linear model, the data acquisition period should be long enough for improving the prediction accuracy. 

### 3.3. Failure Trends by Modules

#### 3.3.1. Failure Rate and NHPP Model Fitting to Modules

The first year and the following trends in membrane failure varied significantly among the modules (Figure A1 in Appendix A). Modules A, B, H, K, N, and O showed a gradual increase in the failure rate, even though some of them showed fluctuation. On the contrary, Modules F, G, I, J, L, and M showed rapidly increasing trends. Others showed very small or almost no increase in the failure rate (Modules C, D, E). This substantial variation among the failure rates of the modules was probably due to the variation in the hollow fibers’ properties in their manufacturing process. Therefore, the fitting and prediction of failure rates with the NHPP models were unsuccessful for each of the modules, while the variations among the modules were averaged for the whole plant, making it possible to apply the NHPP models to them (Figure 3). Consequently, a significant deviation in the fitted model parameters of each module was derived, as shown in Table 4. Module L showed noticeably higher failure rates that were close to the permissible failure rate of 22 membranes/year, which corresponds to LRV 2. Such a high failure rate suggested the necessity of module replacement to maintain the filtration performance of the system.

**Figure A2:**
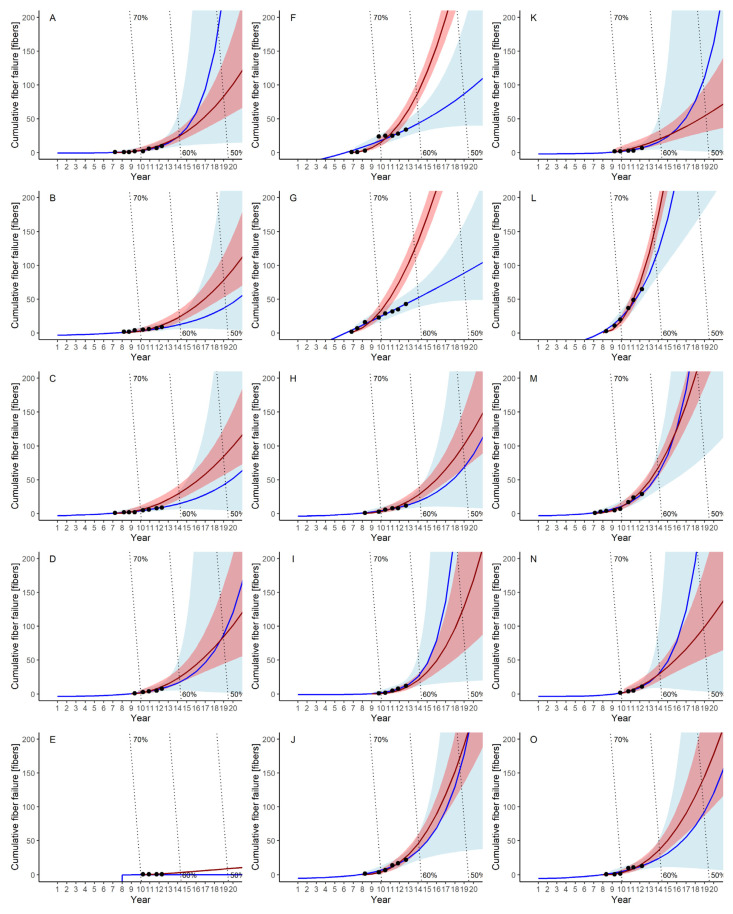
Cumulative failure for each membrane module. Train 1: (**A**–**E**); Train 2: (**F**–**J**); and Train 3: (**K**–**O**). Solid lines indicate fitted curves and highlighted areas show 95% confidence intervals for the power law NHPP model (red) and log-linear NHPP model (blue).

The actual cumulative fiber failure trends also varied significantly by module (Figure A2), leading to the different trends in the cumulative failure curves predicted by the power law and log-linear models. In Module G, in which the cumulative fiber failure showed an approximately linear increasing trend, the power law model (red line) hardly fitted the actual data, while the log linear model (blue line) fit well. This difference is due to the model structures of cumulative fiber failure N(t) of these two NHPP models (Equations (3) and (10)). The power law model follows the power of the operation time t and shows the exponential growth of cumulative failure numbers, resulting in lower fitting and prediction performance. Furthermore, the cumulative failure curves predicted by the power law model (red lines) are above those by the log-linear model (blue lines) in Modules B, C, and F, while the predicted curves by power law model are below those by the log-linear model in Modules A, I, K, and N. 

The 95% confidence intervals for the log-linear model are always wider than those for the power law model, suggesting the larger variability in the failure predicted by the log-linear model than the power law model, which is in agreement with the AIC values shown in Table 4. This indicates that, if the degree of model fitting of both NHPP models is similar, the power law model can provide more accurate prediction performance. Thus, in the failure prediction of individual modules, both NHPP models should be compared in terms of the degree of fitting and the confidence intervals of the prediction. 

#### 3.3.2. Bootstrap Estimation of Model Parameters

**Figure A3:**
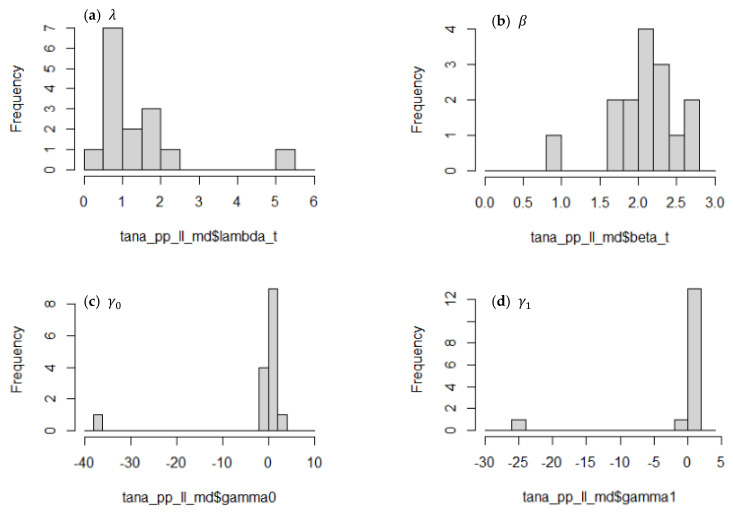
Histogram of estimated parameters for each module.

The model parameters of both NHPP models for each module were significantly varied, as shown in the previous section (Table 4). In some modules, the trends in the predicted cumulative failure curves by the power law and log-linear models were significantly different (Figure A2). These variations pose difficulties in predicting the failure trends of and determining when and which module should be replaced. To improve the prediction accuracy of cumulative membrane failure per module, a bootstrap method was applied to the model parameters for both the power law and the log-linear models. Due to the wide range of variations for the in fitted model parameters (Table 4, Figure A3), the median value for each parameter was used to estimate the distributions of parameters.

**Figure 4:**
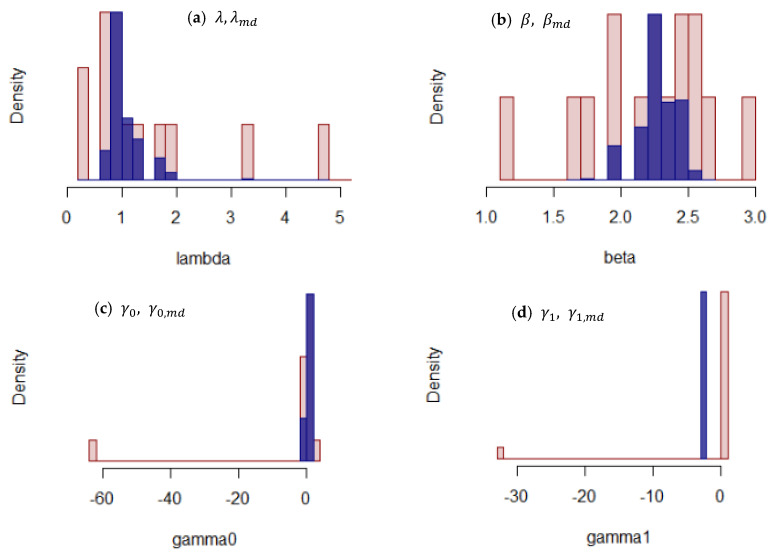
Distribution of the estimated model parameters for each module (red) and model parameter medians ($\lambda_{md}$, $\beta_{md}$, $\gamma_{0,md}$, $\gamma_{1,md}$) estimated by 10,000 bootstraps (blue).

The bootstrapped distribution of median of each model parameter (10,000 iterations) as well as the model parameter for each module are shown in Figure 4. The widely distributed power law model parameters, λ and β, for each module indicate the significant difference in the failure trend due to the variations in the properties of the membranes, as mentioned in the previous section. Distributions of bootstrapped parameters, $\lambda_{md}$ and $\beta_{md}$, and their 95% confidence intervals show the range of medians of these model parameters, which represent the overall trends (Figure 4, Table 4). The bootstrapped median value for λ was 0.95, which is significantly different from the average of the estimated value for each module, 1.73 for λ (Table 4); this is attributed to the extreme parameter values of Modules G and L. The failure trend of Module G was linearly increasing, which could not be fitted by the power law model, and Module L showed a rapidly increasing trend of cumulative failure (Figure A2). Although these failure trends are different, they probably led to larger λ values by the power law model. Conversely, the bootstrapped median value and the average value for β were 2.28 and 2.20, respectively, which are within a similar range. 

The distributions of log-liner model parameters, $\gamma_{0}$ and $\gamma_{1}$, contained the extreme values (Figure 4c,d, Table 4), which significantly influenced the arithmetic means of those parameters. Thus, the averages of the estimated values of −3.83 and −1.88 for $\gamma_{0}$ and $\gamma_{1}$, respectively, are significantly different from the bootstrapped median values of 0.26 and 0.20 for $\gamma_{0,md}$ and $\gamma_{1,md}$, respectively (Table 4). This difference is apparently due to the significantly smaller parameter values for Module E, in which only one membrane failure was observed during the operation. This indicates that the log-linear model produced an extreme response to the very small number of failures due to its exponential form of the intensity function (Equation (11)). By taking the bootstrapped median, the influence of the extreme values can be eliminated, while the variations in the properties of the modules were incorporated.

**Figure 5:**
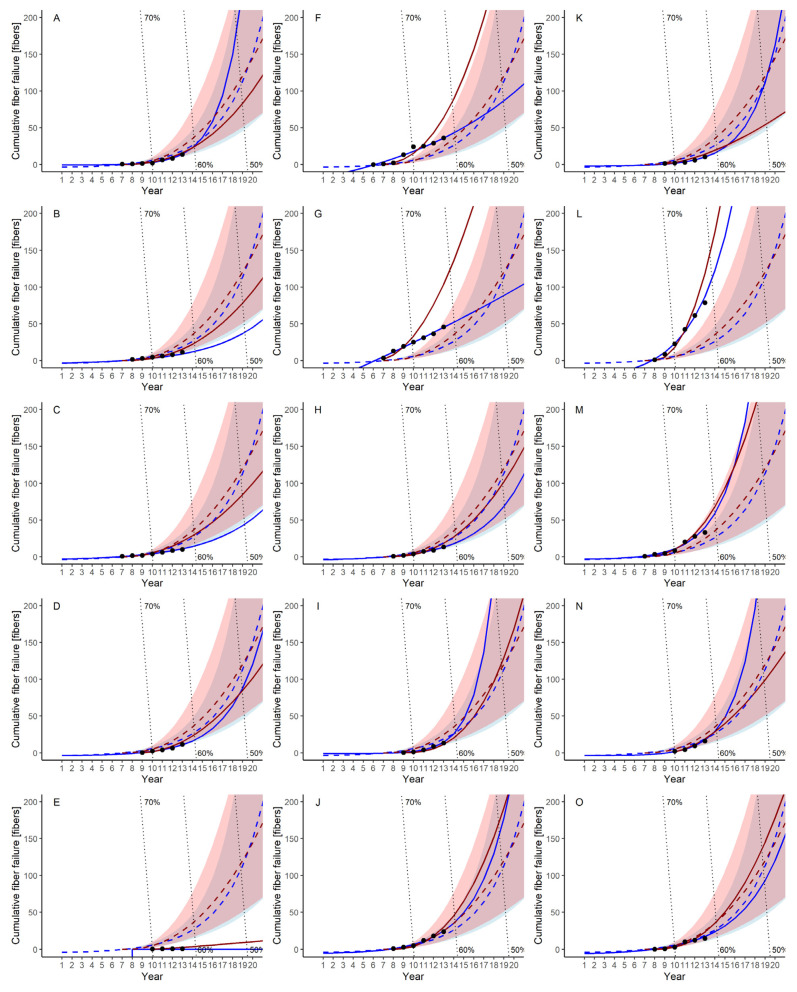
Cumulative failure for each membrane module. Train 1: (**A**–**E**), Train 2: (**F**–**J**), and Train 3: (**K**–**O**). Solid lines indicate curves fit by the power law NHPP model (red) and the log-linear NHPP model (blue), and dashed lines indicate the curves predicted from bootstrapped parameters.

The cumulative failure curves predicted using the bootstrapped parameters (hereafter, the bootstrapped cumulative failure curves) for both the power law and log-linear models and their 95% confidence intervals are shown in Figure 5, along with the curves predicted for each module by the power law and log-linear models. The bootstrapped cumulative failure curve of power law model (dashed red line) and that of the log-linear model (dashed blue line) show similar trends, as indicated by the cumulative failure curves predicted using the 13-year data (Figure 3e). The advantage of the bootstrapped cumulative failure curves is that they can estimate the confidence intervals of the cumulative failure curve of each module. 

## 4. Discussion

### 4.1. Criteria for Membrane Replacement

We proposed two criteria for membrane module replacement: the membrane failure rate and the reduction in membrane performance. Although these two criteria used to be reported independently, to the best of our knowledge, we are the first to show how to combine these two criteria.

#### 4.1.1. Replacement of the Membrane Module by Failure Rate

**Figure 6:**
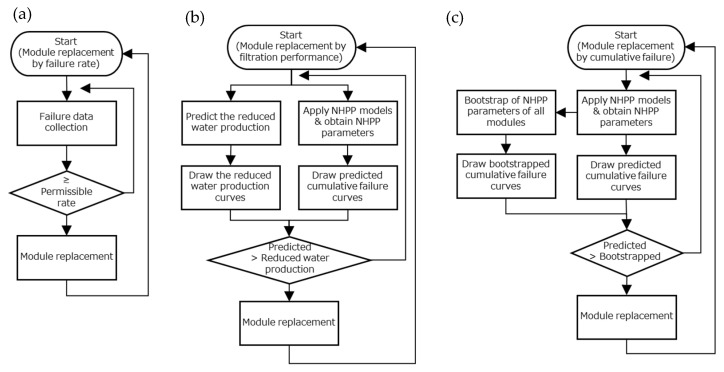
Flow charts of membrane module replacement. (**a**) replacement by failure rate; (**b**) replacement by filtration performance; and (**c**) replacement by cumulative failure.

A criterion of 330 membrane fiber failures per year for a whole plant, which corresponds to LRV 2, was proposed for module replacement (Figure 6a). If the number of failures of the whole plant exceeds this criterion, all modules in the plant should be replaced. Practically, the prediction of the failure rate in the forthcoming one or two years is important for waterworks to know the likelihood of module replacement, as one or two years is required to allocate budget. This criterion is subject to the raw water quality and the expected treatment efficiency of membrane filtration systems, and, thus, it should be determined individually for each membrane filtration plant.

#### 4.1.2. Replacement of the Membrane Module by Filtration Performance

The reduction in filtration performance is estimated from two factors: the observed flux reduction due to membrane fouling and the reduction in membrane surface area caused by the plugging of failed membrane fibers (Figure 6b). The predicted cumulative failure curves by power law and log-linear models, as well as performance reduction levels, are shown in Figure 3.

The cumulative failure curve predicted from the data up to the 13th year by the power law model crosses the performance level curve of 60% in the 14th year of operation. This indicates that all modules should be replaced in the 14th year if the performance level below 60% of the initial level is not permissible. If the permissible performance level is set to 50%, the predicted curve crosses the performance level curve of 50% in the 18th year, which indicates a longer service life of the membrane modules. However, the predicted failure rate curve exceeds the permissible level in the 17th year, as explained in the previous section. Thus, in this case, the failure rate criterion should be preferred.

In the case of log-linear model curves from the data up to the 13th year of operation, a similar decision would be made as with the power law model. However, careful attention should be paid to the prediction from the log-linear model curves using a smaller number of data up to the 9th, 10th, or 11th years, as they might lead to the overestimation of cumulative failures. 

### 4.2. Comparison between the Power Law and Log-Linear Models

The difference between the cumulative failure curves predicted by the power law model and those by the log-linear model are quite large up to the 11th year of operation, and the predicted cumulative failure from the log-linear model is significantly greater than that predicted by the power law model (Figure 3). As mentioned in Section 3.2.2, the AIC values of the power law model that are smaller than those of the log-linear mode indicate that the power law model is superior for failure prediction than the log-linear model in this study. However, the RMSE values of the log-linear model after 12 years are smaller than those of the power law model, which indicates a better fit of the log-linear model than the power law model to the membrane failure data. However, in a study on the application of the power law and the log-linear models for prediction of a water main failure rate, the log-linear model was selected based on log-likelihood comparison [44]. Thus, it is recommended to apply both NHPP models to the data obtained in different membrane filtration plants, and compare their performance of membrane failure prediction to select the most suitable model with superior performance.

### 4.3. Membrane Module Replacement Strategy

In a large-scale membrane filtration plant, it may be necessary to replace some of the membrane modules earlier than others because all modules cannot be replaced at one time [16]. Thus, it is important to predict both the failure rate and cumulative failure number for each membrane module to select the membrane modules for earlier replacement than others. 

This paper proposed a new strategy for determining when and which module should be replaced. In this strategy, the year of membrane module replacement is determined by comparing the actual failure data and the predicted cumulative failure curves with their confidence interval by the bootstrap method. As described in Section 3.3.2, the bootstrapped cumulative failure curve with its confidence interval showed the overall cumulative failure trend together with variations among the modules due to the property variations of membrane fibers. Thus, the bootstrapped curve could be helpful for the selection of modules to be replaced earlier than others. 

The proposed procedure (Figure 6c) is as follows:

(1) Estimate the NHPP model parameters for each module from the actual failure data, and draw the predicted cumulative failure curve from the estimated parameters of the NHPP models.
(2) Obtain the bootstrapped median and its 95% confidence interval of model parameter for each module, and draw the bootstrapped cumulative failure curve with a confidence interval.
(3) Compare the predicted cumulative failure curve and the bootstrapped cumulative failure curve, and select the modules to be replaced when the predicted curve is above the upper boundary of the confidence interval of the bootstrapped curve.

**Figure A4:**
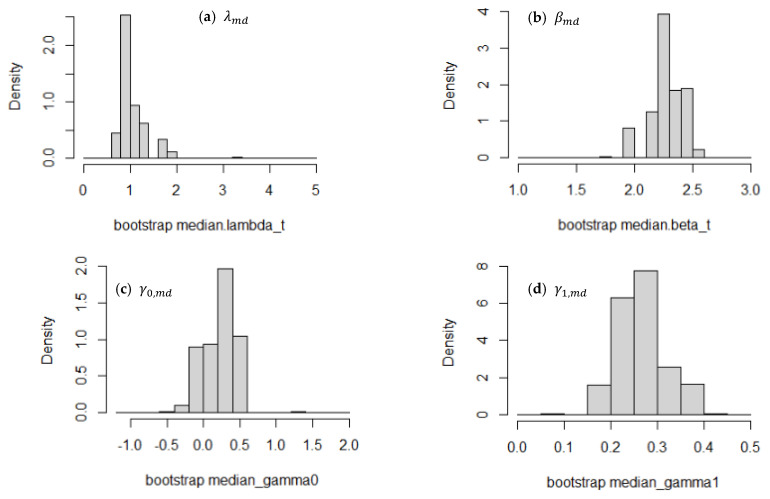
Distribution of model parameter medians ($\lambda_{md}$, $\beta_{md}$, $\gamma_{0,md}$, $\gamma_{1,md}$) estimated by 10,000 bootstrap times.

If the trend of the actual cumulative failure and/or the predicted failure curve of a certain membrane module is above the upper boundary of the confidence interval of the bootstrapped cumulative failure curve, the module is assumed to have a significantly greater number of failed fibers than others. Modules F, G, L, and M could be categorized as such modules because the failure curves predicted by the power law model or by the log-linear model are above the bootstrapped cumulative failure curves and the upper boundary of the confidence interval (Figure 5). Among them, Modules F, G, and M will be subject to replacement to maintain the safety of the system, although their failure rates were below the permissible limit (LRV 2) in the 13th year of operation (Figure A1). For Module L, the replacement would be determined according to either of its high cumulative failure trend or its high failure rate, which were close to the permissible level, as explained in Section 3.3.1. Careful attention to Modules I, J, and N is needed because at least one of the failure curves predicted by the NHPP models will exceed the upper boundary of the confidence interval of the bootstrapped cumulative failure curve in the near future. Other modules with lower cumulative failure numbers could be continuously used for a period longer than 13 years. The predicted membrane failure trends shown in Figure 5 should be updated every year to reliably predict membrane failures. Distribution of model parameter medians was depicted in Figure A4.

The membrane failure data used in this study are specific to the membrane modules used in the membrane filtration plant of this study. Thus, the membrane failure rates and trends might be different in other plants with membrane modules different from those in this study. However, the proposed approach could be applied to the failure prediction of different membrane filtration plants using different membrane modules, as it requires only the membrane failure data. Accordingly, the proposed approach could be also applied to the failure prediction of other membrane filtration plants of wastewater treatment, water reuse, or gas/oil treatment systems in which the membrane replacement is determined in a similar manner with a water purification system [44,45]. 

## 5. Conclusions

Because of ageing, the probability of membrane failure changes with membrane filtration plant operation over time. Therefore, we proposed two nonhomogeneous Poisson process (NHPP) models, namely, the power law model and the log-linear model, to predict the number of membrane failures in an actual membrane filtration plant. The methods to apply these NHPP models to the membrane failure data obtained from a full-scale plant were delineated. 

The power law model showed lower AIC values than the log-linear model in predicting the membrane failures of the plant. In addition, the log-linear model showed overestimating tendency and wider 95% confidence ranges, especially with short operational periods to acquire failure data, suggesting its lower prediction accuracy compared to the power law model. Although the failure trends predicted by both models converged within a small range when the operational period of failure data acquisition extended, the power low model was found to be the preferred model for predicting the membrane failure trend of the whole plant. 

To estimate the year of membrane replacement in a water purification plant, two criteria, i.e., the membrane failure rate and the membrane performance reduction, were proposed, and their use was verified using the actual membrane failure data. The membrane failure rate was set based on the microbial safety of more than a 2-log reduction. The reduction levels of membrane permeability were set to between 50% and 70% of the initial flux. Combining these two criteria, it is possible to integrate membrane replacement strategies based on filtered water quality and the filtration performance. Using the membrane failure data obtained in this study, it was demonstrated that membrane service life before replacement is 14 to 17 years depending on the selection of the performance level. 

The trends in the failure rates per module, as well as the cumulative failure numbers, varied significantly due to the variations in the properties of the modules, probably resulting from material variations during membrane production. Thus, it might be better to replace those membrane modules with higher failure rates than others in order to save the cost of membrane replacement. Thus, a bootstrap method was employed to consider the property variation in the membrane modules in the NHPP models for failure prediction, which successfully simulated the overall cumulative failure trend of a module using bootstrapped median parameters and their 95% confidence intervals. It is suggested to replace a membrane module if the predicted failure trend is higher than the upper confidence boundary of bootstrapped failure curve. In the case of the membrane modules examined in this study, 4 out of 15 modules were selected to be replaced earlier than others.

Until now, the determination of module replacement has been dependent on the experience and knowledge of seasoned waterworks staff. However, the strategy for membrane module replacement proposed in this study provides a systematic framework for membrane replacement based only on the failure data observed in each membrane filtration plant, which provides support to the plant operators and waterworks staff.

## Figures and Tables

**Table 1:** Overview of the membrane filtration plant.

	Operational Parameter
Raw water	Stream water
Process flow	Intake → Receiving pond → Membrane filtration → Distribution pond
System	3 trains (5 module/train)
Capacity	350 m^3^/day
Filtration flux	1.28 m^3^/m^2^/day
Membrane cleaning	Backwash: Once in 45 min (air 30 s + water 30 s)
Operating time	Chemical cleaning: every 6 to 9 months (acid, hypochlorite)

**Table 2:** Specifications of the hollow fiber membrane.

	Specification
Molecular weight cut off (MWCO)	150,000
Material	Polyacrylonitrile (PAN)
Filtration mode	Dead end mode (outside-in)
Length	ca. 1900 mm
Inner/outer diameter	0.9 mm/1.25 mm
Number of membranes per module	ca. 5000 fibers

**Table 3:** Estimated parameters for power law model and log-linear model intensity functions.

Years of Operation	Power Law Model					Log-Linear Model				
	λ	β	AIC	RMSE		$\gamma_{0}$	$\gamma_{1}$	AIC	RMSE	
				Failure Rate	Cumulative Failure				Failure Rate	Cumulative Failure
~9	7.57	2.35	−546	12.2	26.9	1.89	0.66	−271	259.2	284.9
~10	6.58	2.51	−1400	21.4	22.0	2.09	0.54	−571	117.5	123.9
~11	5.68	2.64	−2970	28.0	25.1	2.25	0.46	−1038	69.3	66.8
~12	5.59	2.66	−5248	28.4	25.5	2.58	0.33	−1446	24.5	15.6
~13	5.57	2.66	−8427	28.5	25.5	2.75	0.27	−1977	13.4	11.2

**Table 4:** Estimated parameters for each module.

Module	Power Law Model			Log-Linear Model		
	λ	β	AIC	$\gamma_{0}$	$\gamma_{1}$	AIC
A	0.36	2.53	−37.9	−1.07	0.46	11.5
B	0.64	2.28	−42.1	−0.13	0.18	11.4
C	0.85	2.12	−38.6	−0.13	0.18	14.2
D	1.74	1.96	−20.3	0.22	0.30	8.5
E	0.74	1.19	−	−62.9	−32.2	85.5
F	1.36	2.47	−496.4	1.39	0.07	−38.4
G	4.64	1.97	−716.1	1.83	0.01	−67.8
H	0.78	2.34	−56.9	0.15	0.23	5.5
I	0.33	2.97	−44.9	−0.46	0.55	−1.7
J	0.95	2.55	−163.8	0.53	0.30	−21.7
K	1.90	1.62	−8.2	−0.21	0.37	9.4
L	6.38	2.24	−964.1	2.00	0.27	−202.9
M	0.85	2.61	−313.6	0.31	0.36	−34.0
N	3.36	1.73	−33.7	0.57	0.45	1.5
O	1.01	2.41	−88.0	0.48	0.24	5.0
Average	1.73	2.20		−3.83	−1.88	
Median	0.95	2.28		0.22	0.27	
Bootstrapped median (2.5%, 97.5%)	0.95 (0.78, 1.90)	2.28 (1.97, 2.47)	-	0.26 (−0.21, 0.57)	0.20 (0.18, 0.36)	-

## Data Availability

Not applicable.
